# Recovery Dynamics of Intestinal Bacterial Communities of CCl_4_-Treated Mice with or without Mesenchymal Stem Cell Transplantation over Different Time Points

**DOI:** 10.1155/2020/1673602

**Published:** 2020-10-14

**Authors:** Yanping Xu, Hua Zha, Wenyi Chen, Hongcui Cao, Lanjuan Li

**Affiliations:** ^1^State Key Laboratory for the Diagnosis and Treatment of Infectious Diseases, The First Affiliated Hospital, College of Medicine, Zhejiang University, 79 Qingchun Rd., Hangzhou City 310003, China; ^2^National Clinical Research Center for Infectious Diseases, 79 Qingchun Rd., Hangzhou City 310003, China; ^3^Zhejiang Provincial Key Laboratory for Diagnosis and Treatment of Aging and Physical-Chemical Injury Diseases, 79 Qingchun Rd, Hangzhou City 310003, China

## Abstract

Liver injury has caused significant illness in humans worldwide. The dynamics of intestinal bacterial communities associated with natural recovery and therapy for CCl_4_-treated liver injury remain poorly understood. This study was designed to determine the recovery dynamics of intestinal bacterial communities in CCl_4_-treated mice with or without mesenchymal stem cell transplantation (i.e., MSC and CCl_4_ groups) at 48 h, 1 week (w), and 2 w. MSCs significantly improved the histopathology, survival rate, and intestinal structural integrity in the treated mice. The gut bacterial communities were determined with significant changes in both the MSC and CCl_4_ groups over time, with the greatest difference between the MSC and CCl_4_ groups at 48 h. The liver injury dysbiosis ratio experienced a decrease in the MSC groups and a rise in the CCl_4_ groups over time, suggesting the mice in the MSC group at 48 h and the CCl_4_ group at two weeks were at the least gut microbial dysbiosis status among the corresponding cohorts. Multiple OTUs and functional categories were associated with each of the bacterial communities in the MSC and CCl_4_ groups over time. Among these gut phylotypes, OTU1352_*S24-7* was determined as the vital member in MSC-treated mice at 48 h, while OTU453_*S24-7*, OTU1213_Ruminococcaceae, and OTU841_*Ruminococcus* were determined as the vital phylotypes in CCl_4_-treated mice at two weeks. The relevant findings could assist the diagnosis of the microbial dysbiosis status of intestinal bacterial communities in the CCl_4_-treated cohorts with or without MSC transplantation.

## 1. Introduction

Liver injury is a severe liver condition and has caused significant illness in human worldwide [1, 2]. This condition has been associated with the changes of intestinal microbiota [3, 4]. The intestinal microbiota is involved in the maintenance of the intestinal barrier by several mechanisms such as preventing colonization by pathogenic bacteria and by cooperating with the intestinal epithelium to produce mucin 2 [5]. Recent findings suggest that the disruption of the intestinal barrier is a prerequisite for liver injury [6]. Some probiotics were effective for the prevention of this condition [7, 8]; however, the effective therapies and the corresponding mechanisms remain poorly understood.

The application of mesenchymal stem cells (MSCs) on liver injury repair has attracted increasing attention in recent years. The transplantation of MSCs has been found to alleviate injuries in multiple organs [8–14], including liver injury [15, 16]. Among the MSCs, bone marrow MSCs (BM-MSCs) could improve the clinical indices of liver function in the patients with liver injury caused by hepatitis B [17] and attenuate hepatic ischemia-reperfusion injury in mice [16, 18].

Our previous study has provided important insights on the changes of survival rate, liver biochemistry parameters, histology, and intestinal microbiota between the CCl_4_-treated mice with or without MSC therapy [19]. In the current study, we aimed to (1) determine the recovery dynamics of intestinal bacterial communities of CCl_4_-treated mice with or without MSC transplantation over different time points and (2) investigate the vital phylotypes in the least dysbiotic intestinal bacterial community in CCl_4_-treated mice with or without MSC transplantation over the three time points.

## 2. Materials and Methods

### 2.1. Animal Information

Enhanced green fluorescent protein (GFP) transgenic C57BL/6 mice were purchased from Nanjing Biomedical Research Institute of Nanjing University. Then, female mice were backcrossed to C57BL/6 male mice to produce younger male C57BL/6 mice for isolation and culture of MSCs.

The 6-8*-*week-old male C67BL/6 mice were purchased from Shanghai SLAC Laboratory Animal Co., Ltd. For the induction of acute liver injury (ALI) with CCl_4_. Animals were allowed access to food and water and housed under specific pathogen-free conditions. All animal experimental procedures were conducted according to a protocol approved by the Ethics Committee of the First Affiliated Hospital of Zhejiang University.

### 2.2. Isolation and Culture of Mouse MSCs

MSCs were isolated and cultured as previously described [20]. Briefly, two- to three-week-old wild-type C57BL/6 male mice were sacrificed for obtaining the humeri, tibiae, and femurs, the marrow of which was flushed out thoroughly with 3 ml *α*-minimal essential medium until the bones became pale. The compact bones were chopped into pieces and transferred to collagenase II digestion solution in 15 ml tubes, before being incubated at 37°C for 1.5 h with continuous rotation. The enzyme-treated bone chips were suspended in 7.5 ml C57BL/6-MSC special complete MEM and incubated at 37°C in a 5% CO_2_ incubator. Afterwards, nonadherent cells were removed and the complete MEM was replaced, before harvesting the adherent MSCs using 0.25% trypsin-EDTA and resuspending in fresh complete MEM. Purified MSCs were characterized by inducing osteogenic and adipogenic differentiation and analyzing surface marker expression by flow cytometry (Supplementary Figure S1) [19].

### 2.3. Inducing Acute Liver Injury in Mice with CCl_4_

Sixty 6-8*-*week-old male C67BL/6J mice were intraperitoneally administered with 3 ml/kg CCl_4_ dissolved in olive oil (*v*/*v*, 50%) to induce ALI, while six mice in the negative control (NC) group received olive oil. Six hours after the CCl_4_ administration, mice with ALI were randomly divided into the MSC group (*n* = 30) and the CCl_4_ group (*n* = 30). A 0.1 ml aliquot of PBS containing 5 × 10^5^ MSCs was injected into the tail vein of each mouse in the MSC group, while each mouse in the CCl_4_ group received an injection of 0.1 ml PBS. Mice in the NC group were injected with PBS (*n* = 6). Eighteen mice were randomly selected from the MSC group and anesthetized at 48 h (M48, *n* = 6), 1 w (M1W, *n* = 6), and 2 w (M2W, *n* = 6). Likewise, 18 mice were randomly selected from the CCl_4_ group and anesthetized at 48 h (C48, *n* = 6), 1 w (C1W, *n* = 6), and 2 w (C2W, *n* = 6). The NC group was anesthetized at 48 h.

### 2.4. Tissue Collection and Histopathology

The anesthetized mice were sacrificed for collecting the liver, small intestinal segments, and cecum and colon contents. The liver and small intestinal segments were processed in standard histological methods, while the ileum samples were processed and observed under transmission electron microscopy by a previous study [19].

### 2.5. Molecular Experiments for Illumina Sequencing

DNA was extracted from the cecum and colon contents by using QIAamp DNA stoolMini Kit (Qiagen Inc., USA), before being amplified by fusion dual barcoded primers 319F/806R targeting the V3-V4 regions of bacterial 16SrRNA gene by Dong et al. [19]. The PCR products were purified, quality checked, and subjected for sequencing on an Illumina Miseq sequencer (Illumina Inc. USA) using 2 × 300 bp chemistry.

### 2.6. Intestinal Flora Analyses and Statistical Analyses

#### 2.6.1. Processing of Sequencing Data

Quality filtering, dereplication, chimera filtering, and taxonomy assignment procedures were performed in QIIME software version 1.9.0 as described by Dong et al. [19]. Operational taxonomic units (OTUs) were clustered based on sequence identity threshold ≥ 97%.

#### 2.6.2. Comparisons of Gut Bacterial Communities between the MSC and CCl_4_ Groups at Three Time Points

Permutation analysis of variance (PERMANOVA) was applied to compare the gut bacterial communities in the MSC group and CCl_4_ group at 48 h (M48 versus C48), 1 w (M1W versus C1W), and 2 w (C2W versus M2W) in R software version 3.6.1.

Similarity percentage (SIMPER) analysis was used to compare the dissimilarities of gut bacterial communities between the MSC group and CCl_4_ group at three time points after overall transformation of the dataset in square-root.

Partition Around Medoid (PAM) clustering analysis was performed to cluster all the gut bacterial communities in the CCl_4_ and MSC groups at three time points, after determining the optimal numbers of clusters by using an average silhouette method [21].

#### 2.6.3. Dysbiosis Ratio in the Intestinal Bacterial Communities in the MSC or CCl_4_ Groups

Microbial dysbiosis ratio has been investigated in multiple disease studies to evaluate the microbial dysbiosis status of bacterial community [22–25]. In the present study, the dysbiosis ratio in the gut bacterial communities, i.e., liver injury dysbiosis ratio (LIDR), was defined as the abundance ratio of OTUs associated with the CCl_4_ group and OTUs associated with the NC group at 48 h. A LEfSe analysis was performed to determine the OTUs associated with the CCl_4_ group or NC group. LIDRs of C48 and M48 were compared with a *t*-test, after appropriate data transformation.

#### 2.6.4. Changes of Gut Bacterial Communities of the MSC Groups or CCl_4_ Groups over Time

The LIDRs in M48, M1W, and M2W were compared with one-way ANOVA after appropriate data transformation. *t*-tests were performed for the pairwise comparisons, with Bonferroni's correction for adjusting the *P* values. The LIDRs in C48, C1W, and C2W were compared with the same approaches.

PERMANOVA was used to compare the intestinal bacterial composition between M48, M1W, and M2W, as well as those between M48 and M1W and between M1W and M2W. The same technique was applied for the comparisons of CCl_4_ groups over time.

One-way analysis of variance (ANOVA) was used to compare the alpha diversity, i.e., richness (observed species), diversity (Shannon index), and evenness (Pielou index), in the MSC groups at 48 h, 1 w, and 2 w. *t*-tests were used for the pairwise comparisons, with Bonferroni's correction for the correcting the *P* values. The same approaches were applied for comparisons of alpha diversity of gut bacterial communities of CCl_4_ groups at three time points.

LEfSe analysis was applied to determine the OTUs associated with each of the gut bacterial communities of MSC groups at three time points. The same analysis was carried out for determining the OTUs associated with each of the gut bacterial communities in CCl_4_ groups at three time points.

#### 2.6.5. Changes of Bacterial Networks and Gatekeepers in the MSC or CCl_4_ Groups over Time

Co-occurrence network (CoNet) analysis was carried out to investigate the co-occurrence and coexclusion of OTUs in the MSC groups at 48 h, 1 w, and 2 w. The top 10 OTUs with most correlations at each time point in MSC group were determined. The detailed procedures were performed as described by Wagner Mackenzie et al. [26]. Briefly, Spearman, Pearson, Bray Curtis, Mutual Information, and Kullback-Leibler dissimilarities were chosen to calculate the ensemble inference, with the top 1000 positive and negative correlations recorded. The method-specific *P* values were computed by permutation procedure, followed by a bootstrap step to merge the *P* values into one final *P* value. The same technique was performed for CCl_4_ groups over time.

Gatekeepers were regarded as the OTUs interacting with different parts of the bacterial network that holds together the bacterial community [27]. In the current study, fragmentation was carried out to determine the gatekeeper(s) of the bacterial networks in each of the MSC groups and CCl_4_ groups at different time points. The detailed manipulations were performed as described by Wagner Mackenzie et al. [26]. A total of 10, 000 randomly constructed networks with identical node and edge distributions to the original network was used to create a null distribution of fragmentation scores. Statistical significance was defined as the number of times a fragmentation score greater than that resulting from the removal of the phylotype observed within the null distribution.

#### 2.6.6. Changes of Functional Categories in Gut Microbiota of the MSC or CCl_4_ Groups at Three Time Points

Functional profiles of bacterial communities of MSC groups at three time points were predicted by Tax4fun based in R software [28]. LEfSe analysis was used to determine the functional categories associated with each of the MSC groups at three time points. The same approaches were applied to determine the functional categories associated with each of the CCl_4_ groups at three time points.

## 3. Results

### 3.1. Protective Effects of MSCs against CCl_4_-Induced Liver Injury

**Figure 1:**
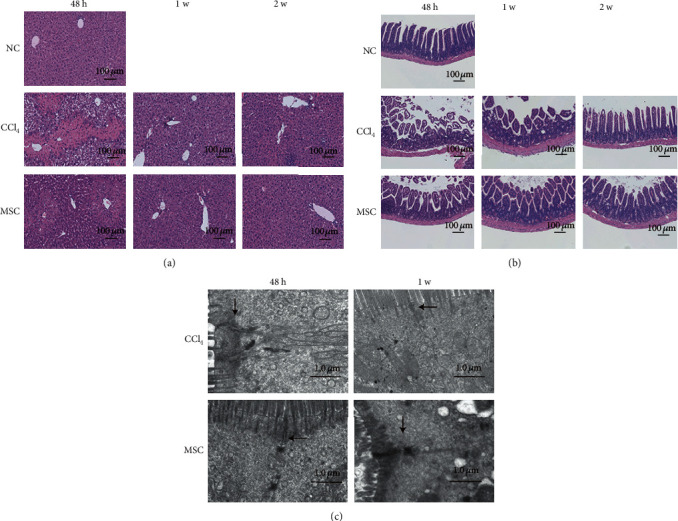
(a) H & E staining of mouse livers sections. Typical signs of steatosis and necrosis of hepatocytes were observed in the liver of the C48 group, while mice in the M48 group experienced a marked improvement. Magnifications: × 20. (b) Hematoxylin and eosin staining of mouse ileum pathology revealed an increase in the length and number of villi in the interposed ileum segment of the M48 group, compared with the C48 group. (c) Ultrastructure of the ileal mucosa using transmission electron microscopy. 48 h, 1 w, and 2 w represent 48 h, 1 week, and 2 weeks after CCl_4_ treatment, respectively.

MSC transplantation dramatically increased the survival rate of CCl_4_-treated mice from 45.5% to 77.3% as described by Dong et al. [19], though there was no significant difference in body weight between the MSC and CCl_4_ groups. The liver and ileum of the MSC transplanted mice have experienced an overall improvement compared with those of the CCl_4_-treated mice (Figures 1(a)–1(c)).

### 3.2. Difference of Intestinal Bacterial Communities between the MSC and CCl_4_ Groups

PERMANOVA revealed that significant difference was determined in the gut bacterial communities between the MSC and CCl_4_ groups at 48 h (*R*^2^ = 0.30, *P* = 0.005) and at 1 w (*R*^2^ = 0.18, *P* = 0.007), but not at 2 w (*R*^2^ = 0.14, *P* > 0.10). SIMPER analyses showed dissimilarity between the MSC and CCl_4_ groups at 48 h (SIMPER dissimilarity = 64.8%) was greater than those at 1 w (SIMPER dissimilarity = 56.7%) and at 2 w (SIMPER dissimilarity = 46.4%).

**Figure 2:**
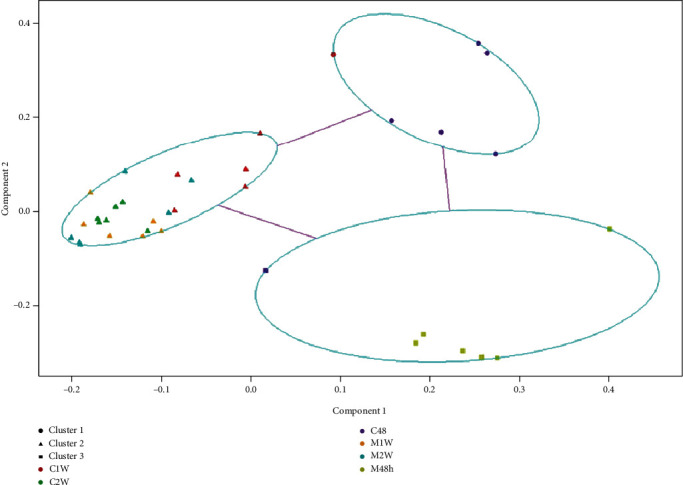
Three clusters of bacterial communities from the MSC and CCl_4_ groups determined by Partition Around Medoid (PAM) clustering analysis. C: carbon tetrachloride- (CCl_4_-) treated group; M: mesenchymal stem cell- (MSC-) transplanted group. 48, 1 w, and 2 w represent 48 h, 1 week, and 2 weeks following CCl_4_ treatment, respectively.

Three and five were determined to be the two optimal numbers for clustering with the highest silhouette scores (Supplementary Figure S2). Three clusters could cluster the bacterial communities of the MSC and CCl_4_ groups compared with five clusters, so three was chosen for PAM analysis. PAM clustering analysis showed three clusters, Cluster_1 (including most C48 and one C1W), Cluster_3 (including all M48 and one C48), and Cluster_2 (including the remaining ones) (Figure 2). All these results suggest that the largest difference in the gut bacterial communities between the CCl_4_ and MSC groups was at 48 h, but not at 1 w and 2 w.

### 3.3. Changes of LIDRs in the MSC or CCl_4_ Groups over Time

LIDRs in the intestinal bacterial communities of all groups were calculated after determining the OTUs associated with the CCl_4_ or NC groups (Supplementary Figure S3). LIDR was significantly lower in C48 (0.15 ± 0.11 SE) than in M48 (12.9 ± 4.37 SE) at baseline (*t*-test, *P* < 0.001).

The LIDRs were significantly different among the M48 (12.9 ± 4.37 SE), M1W (1.83 ± 0.5 SE), and M2W (1.3 ± 0.44 SE) (one-way ANOVA, *P* = 0.002). The LIDR was significantly higher in M48 than in M1W (*t*-test, *P* = 0.01) and M2W (*t*-test, *P* = 0.002), while LIDRs were similar between M1W and M2W (*t*-test, *P* > 0.9). Likewise, the LIDRs were significantly different among the C48 (0.15 ± 0.11 SE), C1W (0.57 ± 0.15 SE), and C2W (1.46 ± 0.13 SE) (one-way ANOVA, *P* = 0.001). The LIDR was significantly higher in C2W than in C48 (*t*-test, *P* < 0.001) and C1W (*t*-test, *P* = 0.001), while the ratios were similar between C48 and C1W (*t*-test, *P* > 0.08).

### 3.4. Changes of Intestinal Bacterial Communities in the MSC or CCl_4_ Groups over Time

PERMANOVA revealed a significant difference among the bacterial communities of M48, M1W, and M2W (*R*^2^ = 0.38, *P* < 0.001). Significant difference was found between M48 and M1W (*R*^2^ = 0.37, *P* = 0.005), but not in M1W and M2W (*R*^2^ = 0.13, *P* > 0.1). Likewise, PERMANOVA showed a significant difference between the C48, C1W, and C2W (*R*^2^ = 0.28, *P* < 0.001). Significant differences were also determined between C48 and C1W (*R*^2^ = 0.15, *P* < 0.04), and between C1W and C2W (*R*^2^ = 0.24, *P* = 0.003).

There was a significant difference in richness among M48, M1W, and M2W (one-way ANVOA, *P* < 0.03). Richness was significantly greater in M48 than in M1W (*t*-test, *P* < 0.03), but similar between M1W and M2W (*t*-test, *P* > 0.2). The diversity and evenness were both similar among M48, M1W, and M2W (one-way ANOVA, *P* > 0.05). By contrast, significant differences were found in diversity (one-way ANVOA, *P* < 0.02) and evenness (one-way ANVOA, *P* < 0.03) between C48, C1W, and C2W. The diversity and evenness were both higher in C2W than in C48 (*t*-test, all *P* < 0.03).

**Figure 3:**
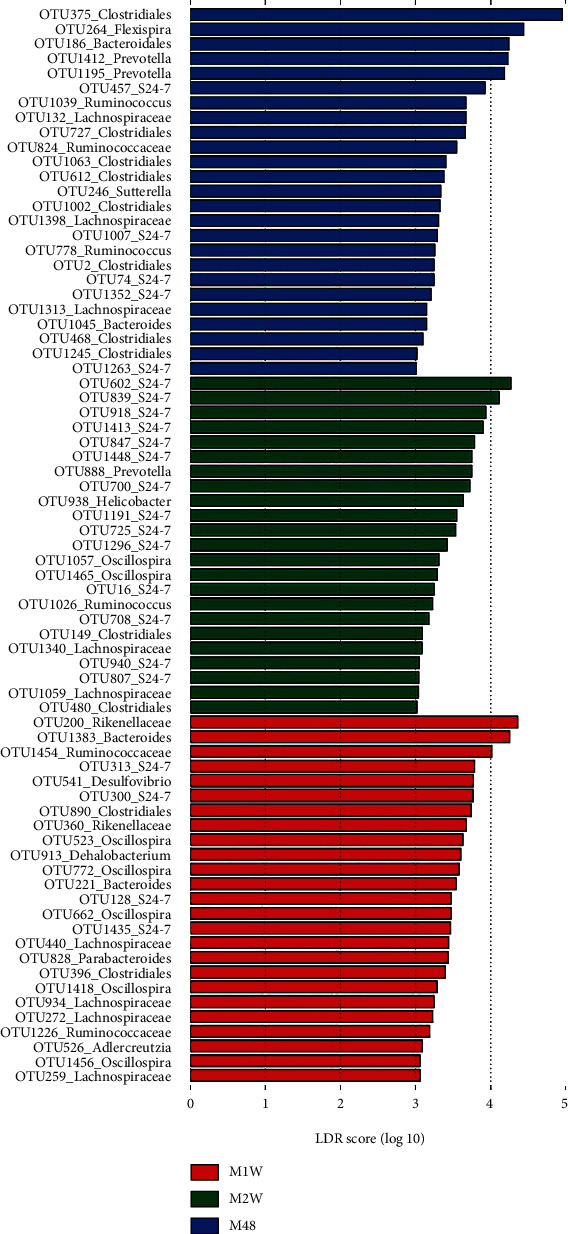
Linear discriminant analysis (LDA) effect size (LEfSe) determined the OTUs associated with each of the three MSC groups at three time points. M: mesenchymal stem cell- (MSC-) transplanted group. 48, 1 w, and 2 w represent 48 h, 1 week, and 2 weeks following CCl_4_ treatment.

A total of 73 OTUs were associated with M48, M1W, or M2W according to LEfSe results. Twenty-five out of the 73 OTUs could distinguish M48 from M1W and M2W, over half of which were assigned to Clostridiales and *S24-7* (Figure 3). A group of 25 OTUs could differentiate M1W from M48 and M2W, over half of which were from *Oscillospira*, Lachnospiraceae, and *S24-7*. The remaining 23 OTUs were more associated with M2W and dominated by OTUs assigned to *S24-7*.

**Figure 4:**
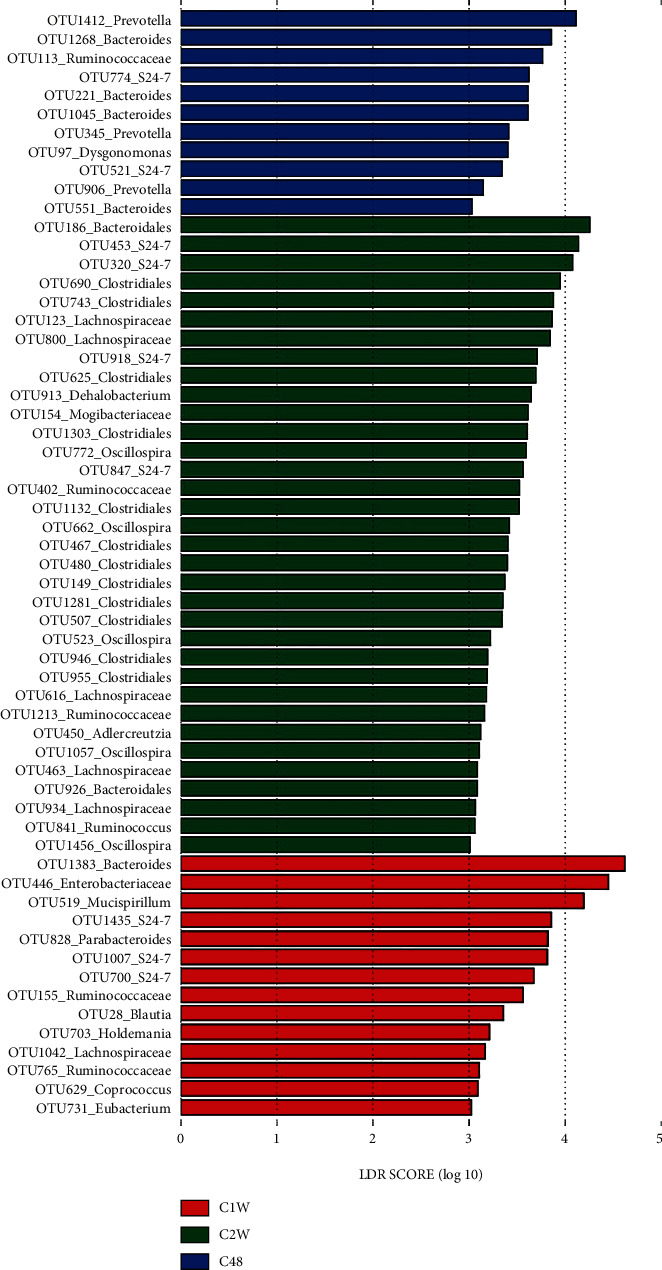
LEfSe analysis determined the OTUs differentiating the gut microbiota from three CCl_4_ groups at three time points. C: carbon tetrachloride- (CCl_4_-) treated group. 48, 1 w, and 2 w indicate 48 h, 1 week, and 2 weeks after performing CCl_4_ administration.

By contrast, 11 OTUs were associated with C48, among which OTUs assigned to *Bacteroides* and *Prevotella* accounted for over half of the phylotypes (Figure 4). Fourteen OTUs from 11 taxa were associated with C1W, with OTU1383_*Bacteroides*, OTU446_Enterobacteriaceae, and OTU519_*Mucispirillum* as the three phylotypes with the largest LDA scores (over 4.0). A total of 34 OTUs were associated with C2W, almost two-thirds of which were from Clostridiales (12 OTUs), Lachnospiraceae (five OTUs), and *Oscillospira* (five OTUs) (Figure 4).

### 3.5. Changes of Networks and Gatekeeper(s) over Time

The bacterial networks belonging to M48, M1W, M2W, C48, C1W, and C2W were determined by CoNet analyses (data not shown). The top 10 OTUs with most correlations in M48, M1W, and M2W were largely distinct (Table 1), with OTUs assigned to *S24-7* being determined in the top 10 OTUs in each of the three networks. Likewise, the top 10 OTUs with most correlations in C48, C1W, and C2W were largely distinct (Table 2), and OTUs assigned to *S24-7* were also determined in the top 10 OTUs in each of the three networks.

Multiple OTUs were determined as gatekeepers in the bacterial networks of M48, M1W, M2W, C48, C1W, or C2W (Table 3). Among them, OTU1352_*S24-7*, i.e., a gatekeeper in the M48 network, was also associated with M48 by LEfSe analysis (Supplementary Figure S4A). Likewise, OTU453_*S24-7*, OTU1213_Ruminococcaceae, and OTU841_*Ruminococcus*, i.e., gatekeepers in C2W, were also associated with C2W determined by LEfSe analysis (Supplementary Figure S4B).

### 3.6. Changes of Functional Categories in Gut Microbiota of the MSC/CCl_4_ Groups over Time

**Figure 5:**
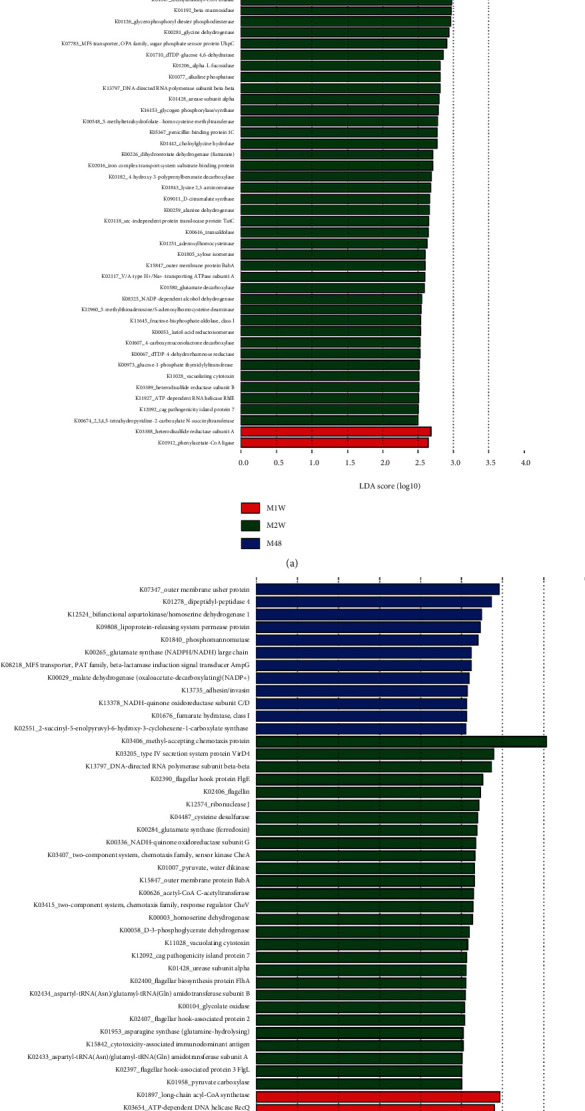
Functional categories associated with (a) MSC groups over time and (b) CCl_4_ groups over time determined by Tax4fun and LEfSe analysis. C: carbon tetrachloride- (CCl_4_-) treated group; M: mesenchymal stem cell- (MSC-) transplanted group. 48, 1 w, and 2 w indicate 48 h, 1 week, and 2 weeks following CCl_4_ treatment, respectively.

Three groups of functional categories were associated with M48 (22), M1W (2), and M2W (42). Glycogen phosphorylase, heterodisulfide reductase subunit A, and hexosaminidase were the three functional categories most associated with M48, M1W, and M2W (Figure 5(a)), respectively. By contrast, outer membrane usher protein, long-chain acyl-CoA synthetase, and methyl-accepting chemotaxis protein were the three functional categories most associated with C48, C1W, and C2W, respectively (Figure 5(b)).

## 4. Discussion

The gut microbiota in CCl_4_-treated mice has been reported in different studies [29–31]. Our previous study provided sufficient findings about the differences between the intestinal bacterial communities in the CCl_4_-treated mice with and without MSC therapy [19]. This study was designed to determine the recovery dynamics of intestinal bacterial communities of CCl_4_-treated mice with or without mesenchymal stem cell transplantation over different time points, which was rarely reported to our limited knowledge.

PERMANOVA, SIMPER, and PAM clustering analyses have been used in a variety of studies [32–34]. In the current study, the differences in bacterial communities between the MSC and CCl_4_ groups at designated times (i.e., 48 h, 1 w, and 2 w) were determined by the three analyses, and the relevant results showed the largest difference between the MSC and CCl_4_ groups occurred at 48 h. As the most obvious difference of survival percentages of mice between the MSC and CCl_4_ groups occurred from 48 h to 1 w, it implied that the change of intestinal bacterial communities was associated with this obvious difference.

PERMANOVA results for MSC groups' comparison suggested the significant change in the gut bacterial communities of MSC groups from 48 h to 1 w, while no such change was found between 1 w and 2 w. By contrast, PERMANOVA results for CCl_4_ groups' comparison suggested the significant changes in gut bacterial communities over the two time periods, i.e., 48 h to 1 w and 1 w to 2 w. An overall difference was determined in the richness of the gut bacterial communities of MSC groups over time, while overall differences were determined in diversity and evenness of C48, C1W, and C2W. These results suggest the recovery mechanisms in gut bacterial communities in the MSC and CCl_4_ groups were different.

The dysbiosis ratio has been investigated in multiple disease studies [22–25]. In this study, LIDR was used to evaluate the dysbiosis status of gut bacterial communities in the MSC and CCl_4_ groups and was significantly lower in C48 than in M48 at baseline. LIDR was decreasing in MSC groups over time, while this ratio experienced an increase in CCl_4_ groups during the same period, which could partly explain why the difference of gut bacterial communities in the MSC and CCl_4_ groups were largest at 48 h. These results implied that MSCs-treated mice experienced the mildest intestinal microbial dysbiosis at 48 h, while the CCl_4_-treated mice had the mildest intestinal microbial dysbiosis at two weeks.

LEfSe results showed different phylotypes were associated with each of the MSC and CCl_4_ groups. Multiple *S24-7* phylotypes were associated with all the MCS and CCl_4_ groups, suggesting different *S24-7* phylotypes could play different roles in each of the six groups. This is consistent with the previous findings, which hold different views on the roles of Bacteroidales *S24-7* in the gut microbiota [35, 36]. Multiple OTUs assigned to Clostridiales were associated with M48, among which OTU375_ Clostridiales was most associated with M48. Likewise, Clostridiales was determined with varied effects on health [37, 38]. OTU186_*Bacteroides* was most associated with C2W in this study. *Bacteroides* species were determined as opportunistic pathogens and probiotics in different studies [39, 40].

The top 10 OTUs with most correlations in the networks of M48, M1W, and M2W were largely different, so as for the CCl_4_ groups, suggesting the gut bacterial networks changed in both the MSC and CCl_4_ groups at different time points. Multiple distinct gatekeepers were determined in the networks of the MSC and CCl_4_ groups over time. Among them, OTU1352_*S24-7* (i.e., a gatekeeper in M48 network) was also associated with M48 by LEfSe analysis, while OTU453_*S24-7*, OTU1213_Ruminococcaceae, and OTU841_*Ruminococcus* (i.e., three gatekeepers in C2W) were also associated with C2W, suggesting these phylotypes could play a key role in maintaining the gut bacterial communities of M48 and C2W (i.e., the cohorts with the highest LIDR in the MSC groups and CCl_4_ groups, respectively). As mentioned above, *S24-7* was determined with varied effects on health. As for the beneficial effect, the enriched *S24-7* in the gut microbiota of mice fed with *Lactobacillus reuteri* ATG-F4 was associated with the improvements of psychological status of a murine model [41]. Ruminococcaceae was believed as a vital member in maintaining the gut health [42], while some *Ruminococcus* species were determined as normal members in gut microbiota [43].

Multiple functional categories were associated with gut microbiota of the MSC and CCl_4_ groups over time in the current study. Among them, glycogen phosphorylase, heterodisulfide reductase subunit A, and hexosaminidase were most associated with M48, M1W, and M2W, respectively, while outer membrane usher protein, long-chain acyl-CoA synthetase, and methyl-accepting chemotaxis protein were most associated with C48, C1W, and C2W, respectively, suggesting these functional categories could play important roles in the changes of gut bacterial communities of the MSC and CCl_4_ groups at different stages. Glycogen phosphorylase normally is located in the brain, liver, and skeletal muscle tissue. Heterodisulfide reductase played an important role in the energy-conserving metabolisms of bacteria and archaea [44, 45]. The serum beta-hexosaminidase level was associated with reticuloendothelial function of the patients with viral hepatitis [46]. *β*-Barrel assembly machine-mediated folding of outer membrane usher protein could be selectively disrupted by nitazoxanide [47]. Long-chain acyl-CoA synthetase in fatty acid metabolism was involved in multiple liver diseases [48]. Methyl-accepting chemotaxis protein was associated with the changes of hepatic inflammation [49].

## 5. Conclusion

In conclusion, the results suggest that the intestinal microbiota, the corresponding networks, and functional categories changed during the recovery dynamics of the MSC or CCl_4_ group. MSC-treated mice were determined with the mildest intestinal microbial dysbiosis at 48 h, with OTU1352_*S24-7* as the vital gut phylotype. CCl_4_-treated mice experienced the least intestinal microbial dysbiosis status at two weeks, with OTU453_*S24-7*, OTU1213_*Ruminococcaceae*, and OTU841_*Ruminococcus* as the vital gut phylotypes. These findings may assist in monitoring the dysbiosis status of intestinal bacterial communities of CCl_4_-treated mice with or without MSC transplantation over different time points.

## Figures and Tables

**Table 1:** The 10 OTUs with most correlations in the bacterial networks of MSC groups at 48 h, 1 w, and 2 w determined by co-occurrence network analysis.

Rank	M48	M1W	M2W
1	OTU1321_Clostridiaceae	OTU191_*S24-7*	OTU865_*S24-7*
2	OTU313_*S24-7*	OTU651_*S24-7*	OTU828_*Parabacteroides*
3	OTU921_*Prevotella*	OTU1268_*Bacteroides*	OTU1288_*S24-7*
4	OTU362_*S24-7*	OTU384_Rikenellaceae	OTU128_*S24-7*
5	OTU229_*Pseudomonas*	OTU143_*S24-7*	OTU344_Clostridiales
6	OTU1305_*Bacteroides*	OTU891_*Oscillospira*	OTU1502_*S24-7*
7	OTU1170_Bacteroidales	OTU717_Streptophyta	OTU718_*Oscillospira*
8	OTU915_*S24-7*	OTU155_Ruminococcaceae	OTU821_*Coprococcus*
9	OTU215_*S24-7*	OTU352_Lachnospiraceae	OTU1052_Lachnospiraceae
10	OTU677_Bacteroidales	OTU727_*S24-7*	OTU952_*S24-7*

Note: M48, M1W, and M2W represent three cohorts of CCl_4_-treated mice receiving MSC transplantation after 48 hours, 1 week, and 2 weeks, respectively.

**Table 2:** The top 10 OTUs with most correlations in the bacterial networks of CCl_4_ groups at 48 h, 1 w, and 2 w (i.e., C48, C1W, and C2W) determined by co-occurrence network analysis.

Rank	C48	C1W	C2W
1	OTU1195_*Prevotella*	OTU143_*S24-7*	OTU1099_AF12
2	OTU421_*Prevotella*	OTU1313_*Lachnospiraceae*	OTU200_*Rikenellaceae*
3	OTU191_*S24-7*	OTU62_Clostridiales	OTU221_*Bacteroides*
4	OTU651_*S24-7*	OTU1127_MVS-40	OTU412_*Rikenellaceae*
5	OTU741_*Parabacteroides*	OTU453_*S24-7*	OTU824_*Ruminococcaceae*
6	OTU264_*Flexispira*	OTU1359_*Koribacteraceae*	OTU804_*S24-7*
7	OTU1453_Clostridiales	OTU521_*S24-7*	OTU1111_Clostridiales
8	OTU483_*Parabacteroides*	OTU1083_MVS-40	OTU656_Clostridiales
9	OTU457_*S24-7*	OTU807_*S24-7*	OTU807_*S24-7*
10	OTU601_*Ruminococcaceae*	OTU246_*Sutterella*	OTU651_*S24-7*

Note: C48, C1W, and C2W represent three cohorts of mice treated with CCl_4_ after 48 hours, 1 week, and 2 weeks, respectively.

**(a):** 

M48	M1W	M2W
OTU1279_Ruminococcaceae	OTU1437_*S24-7*	OTU1132_Clostridiales
OTU1352_*S24-7*		OTU124_Ruminococcaceae
OTU467_Clostridiales		OTU1297_Clostridiales
		OTU1318_*Coprococcus*
		OTU1368_*S24-7*
		OTU1390_*Oscillospira*
		OTU320_*S24-7*
		OTU446_Enterobacteriaceae
		OTU620_Clostridiales
		OTU85_Lachnospiraceae

**(b):** 

C48	C1W	C2W
OTU1195_*Prevotella*	OTU123_Lachnospiraceae	OTU104_Ruminococcaceae
OTU1039_*Ruminococcus*	OTU1284_Lachnospiraceae	OTU440_Lachnospiraceae
OTU1202_Clostridiales	OTU1340_Lachnospiraceae	OTU1085_Ignavibacteriaceae
OTU1275_*S24-7*		OTU1213_Ruminococcaceae
OTU1340_Lachnospiraceae		OTU1383_*Bacteroides*
OTU1383_*Bacteroides*		OTU1450_*Adlercreutzia*
OTU1474_Mogibacteriaceae		OTU15_Lachnospiraceae
OTU311_*S24-7*		OTU279_Rikenellaceae
OTU421_*Prevotella*		OTU453_*S24-7*
OTU572_Lachnospiraceae		OTU62_Clostridiales
OTU577_*S24-7*		OTU841_*Ruminococcus*
OTU637_*Staphylococcus*		
OTU650_*S24-7*		
OTU703_*Holdemania*		
OTU918_*S24-7*		

## Data Availability

The raw sequencing data were deposited in NCBI under BioProject accession no. PRJNA660814 and will be available once the manuscript is accepted.

## Abbreviations

MSCs: Mesenchymal stem cells
CCl_4_: Carbon tetrachloride
GFP: Green fluorescent protein
ALI: Acute liver injury
PBS: Phosphate-buffered solution
OTUs: Operational taxonomic units
LEfSe: Linear discriminant analysis (LDA) effect size
PERMANOVA: Permutation analysis of variance
SIMPER: Similarity percentage analysis
PAM: Partition Around Medoid clustering analysis
ANOVA: One-way analysis of variance
CoNet: Co-occurrence network
LIDR: Liver injury dysbiosis ratio.

## Supplementary Materials

Supplementary Materials Figure S1: phenotype and differentiation of bone marrow-derived- (BM-) MSCs. (A) Fluorescence-activated cell sorting results showed that mesenchymal stem cells (MSCs) were positive for CD44 (99.5%), Sca-1 (98.9%), and CD29 (98.6%), but negative for CD45 (1.7%), CD11b (1.51%), CD31 (1.30%), and MHC-1a (1.2%). (B) C57BL/6 MSCs showed classic spindle-shaped morphology. (C) Differentiation of MSCs into osteocytes (×10) by staining with Alizarin Red S. (D) Differentiation of MSCs into adipocytes (×20) by staining with Oil Red O. Figure S2: optimal cluster numbers determined by average silhouette analysis. Figure S3: identification of OTUs associated with the CCl_4_ and negative control (NC) groups by linear discriminant analysis (LDA) effect size (LEfSe). Figure S4: the vital phylotypes in the (A) M48 and (B) C2W groups determined by Venny program, based on the LEfSe results and gatekeepers determined by fragmentation analysis.
