# Supplementary material for: Recovery Dynamics of Intestinal Bacterial Communities of CCl4-Treated Mice with or without Mesenchymal Stem Cell Transplantation over Different Time Points
Source: Biomed Res Int. 2020 Oct 14;2020:1673602. doi: 10.1155/2020/1673602 (PMC7584945; doi:10.1155/2020/1673602)

**Supplementary Material**

**Recovery dynamics of intestinal** **bacterial communities of CCl_4_- treated mice with** **or without mesenchymal stem cells transplantation** **over different time points**

Yanping Xu^1,2†^, Hua Zha^1,2†^, Wenyi Chen^1,2^, Hongcui Cao†^1,2,3^**^*^**, Lanjuan Li^1,2^

^1^ State Key Laboratory for the Diagnosis and Treatment of Infectious Diseases, The First Affiliated Hospital, College of Medicine, Zhejiang University, 79 Qingchun Rd., Hangzhou City 310003, China

^2^ National Clinical Research Center for Infectious Diseases, 79 Qingchun Rd., Hangzhou City 310003, China

^3^ Zhejiang Provincial Key Laboratory for Diagnosis and Treatment of Aging and Physic-chemical

Injury Diseases, 79 Qingchun Rd, Hangzhou City 310003, China

**^*^ Correspondence:**

Hongcui Cao, M.D.

State Key Laboratory for the Diagnosis and Treatment of Infectious Diseases, The First Affiliated Hospital, College of Medicine, Zhejiang University; Collaborative Innovation Center for the Diagnosis and Treatment of Infectious Diseases, 79 Qingchun Rd., Hangzhou City 310003, China. Tel: 86-571-87236451; Fax: 86-571-87236459

E-mail: hccao@zju.edu.cn

Figure S1: Phenotype and differentiation of bone marrow-derived (BM)-MSCs. (A) Fluorescence-activated cell sorting results showed that mesenchymal stem cells (MSCs) were positive for CD44 (99.5%), Sca-1 (98.9%) and CD29 (98.6%), but negative for CD45 (1.7%), CD11b (1.51%), CD31 (1.30%) and MHC-1a (1.2%). (B) C57BL/6 MSCs showed classic spindle-shaped morphology. (C) Differentiation of MSCs into osteocytes (×10) by staining with Alizarin Red S. (D) Differentiation of MSCs into adipocytes (×20) by staining with Oil Red O.


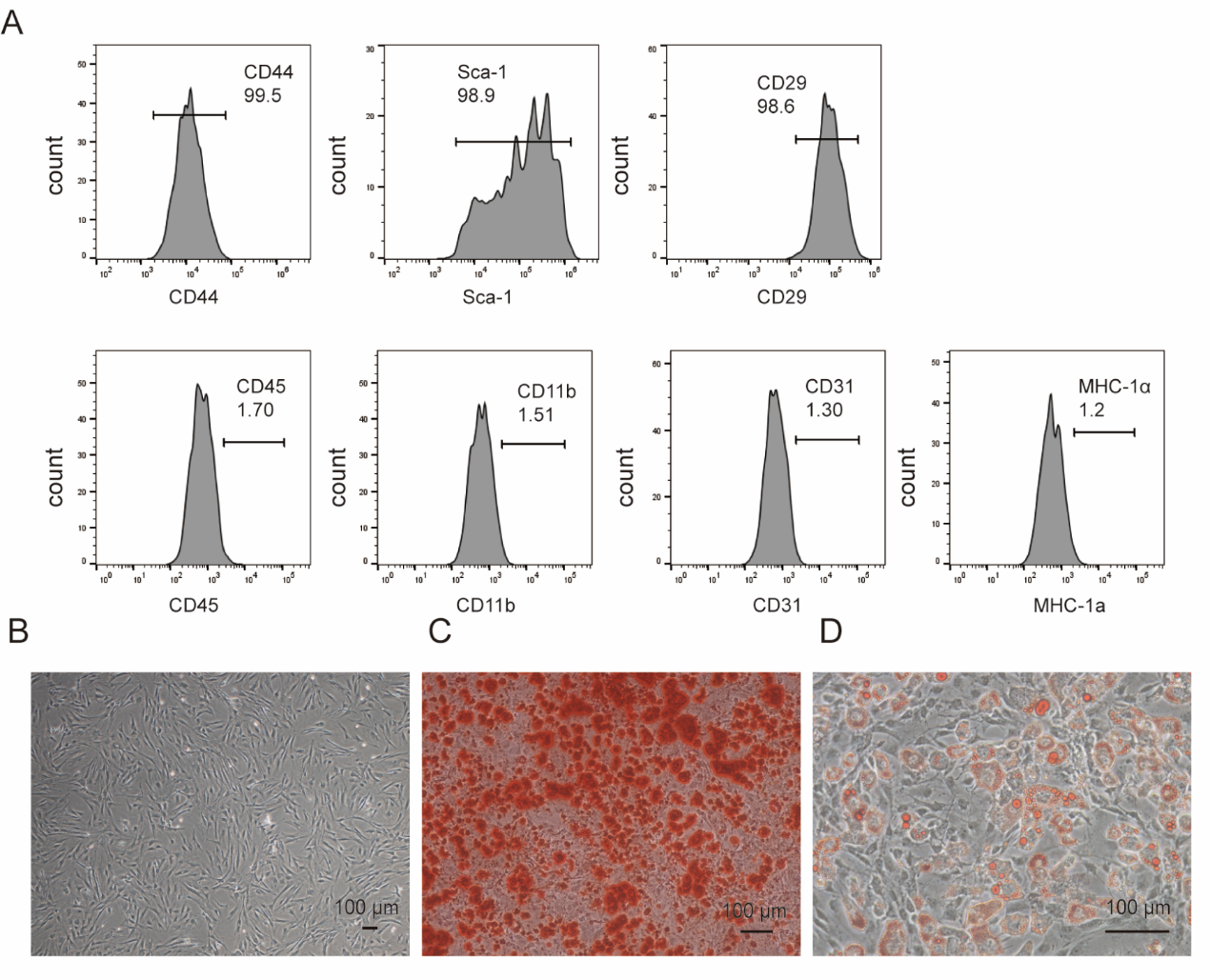


Figure S2: Optimal cluster numbers determined by average silhouette analysis.


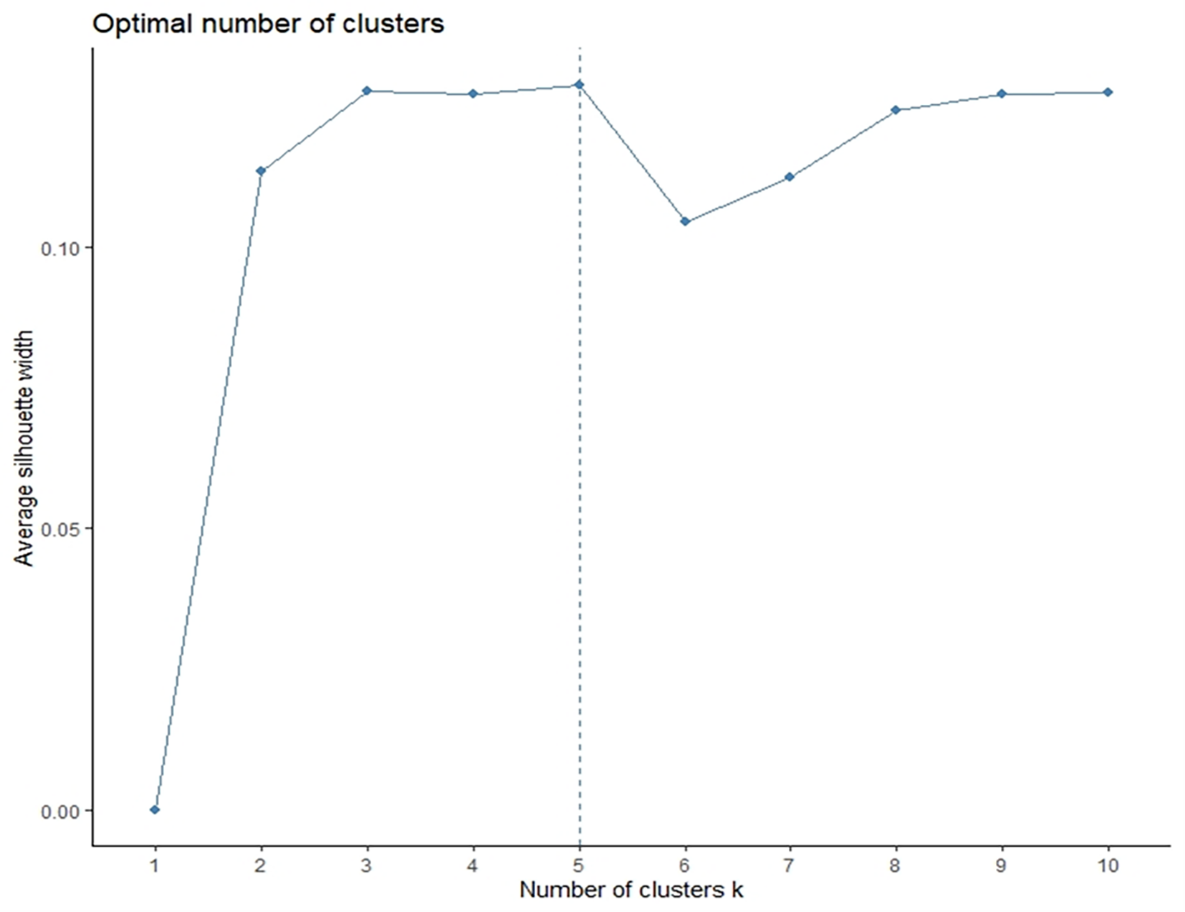


Figure S3: Identification of OTUs associated with CCl_4_ and negative control (NC) groups by linear discriminant analysis (LDA) effect size (LEfSe).


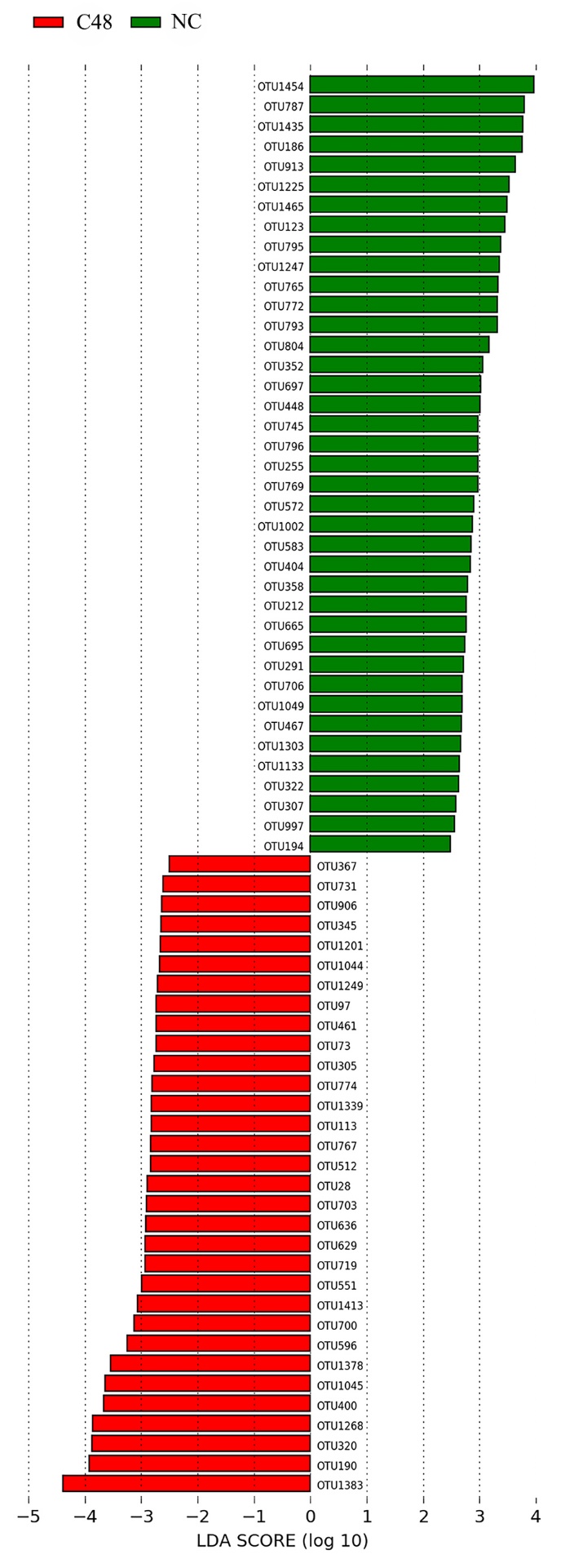


Figure S4: The vital phylotypes in the (A) M48 and (B) C2W groups determined by Venny program, based on the LEfSe results and gatekeepers determined by fragmentation analysis.

A


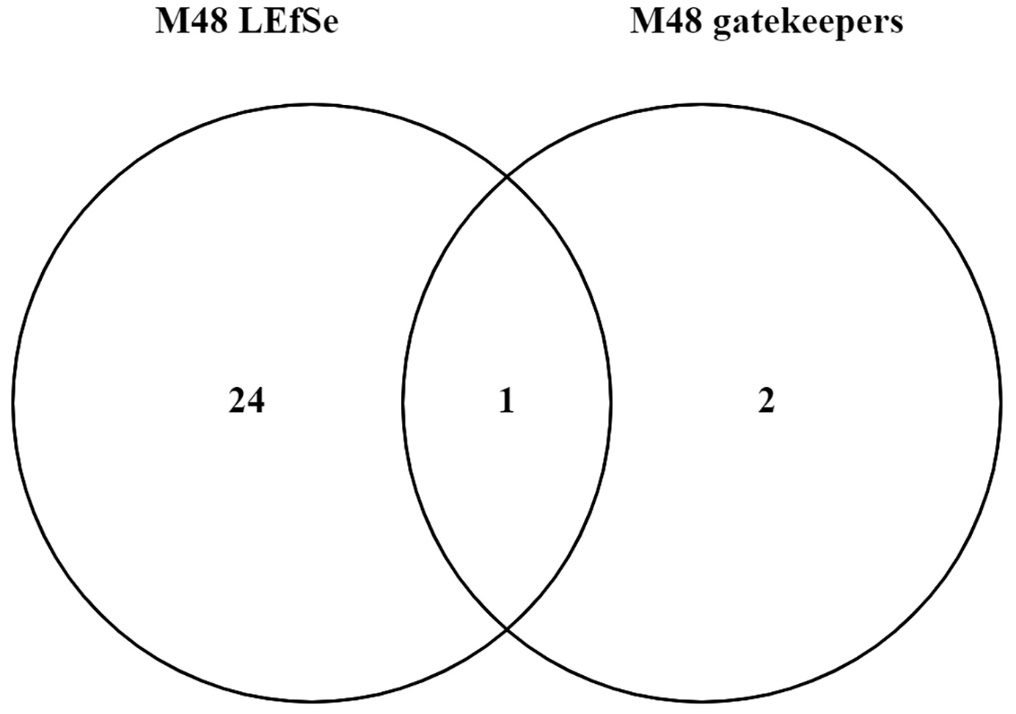


B


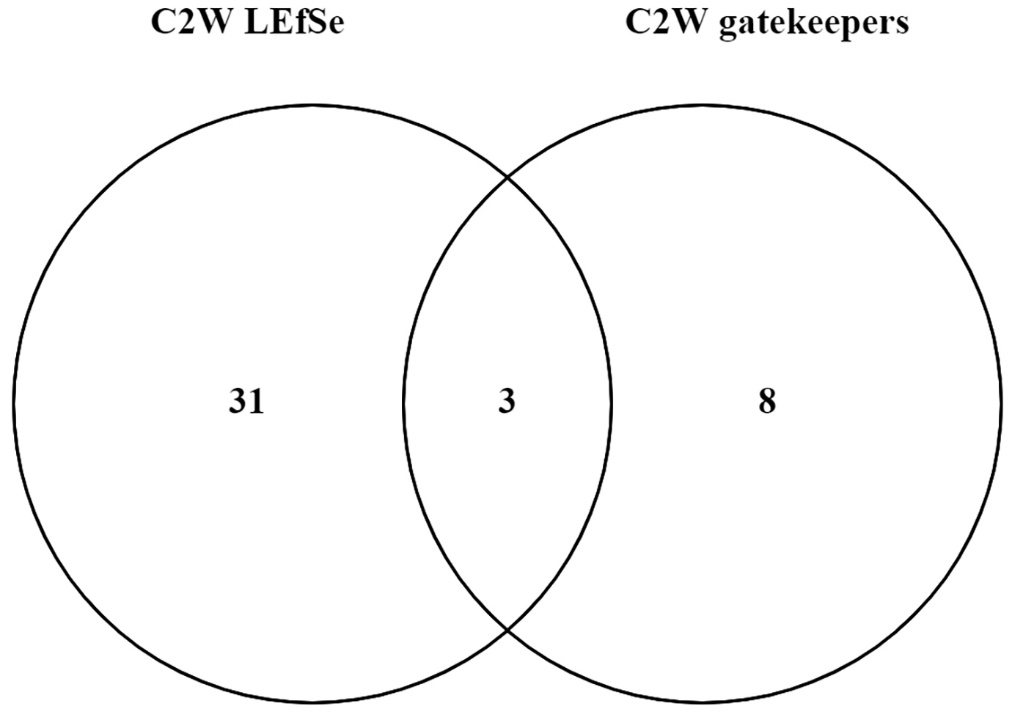

Supplement: Supplementary Materials — Figure S1: phenotype and differentiation of bone marrow-derived- (BM-) MSCs. (A) Fluorescence-activated cell sorting results showed that mesenchymal stem cells (MSCs) were positive for CD44 (99.5%), Sca-1 (98.9%), and CD29 (98.6%), but negative for CD45 (1.7%), CD11b (1.51%), CD31 (1.30%), and MHC-1a (1.2%). (B) C57BL/6 MSCs showed classic spindle-shaped morphology. (C) Differentiation of MSCs into osteocytes (×10) by staining with Alizarin Red S. (D) Differentiation of MSCs into adipocytes (×20) by staining with Oil Red O. Figure S2: optimal cluster numbers determined by average silhouette analysis. Figure S3: identification of OTUs associated with the CCl4 and negative control (NC) groups by linear discriminant analysis (LDA) effect size (LEfSe). Figure S4: the vital phylotypes in the (A) M48 and (B) C2W groups determined by Venny program, based on the LEfSe results and gatekeepers determined by fragmentation analysis. [file 1673602.f1.docx]
